# ESBL Detection: Comparison of a Commercially Available Chromogenic Test for Third Generation Cephalosporine Resistance and Automated Susceptibility Testing in *Enterobactericeae*

**DOI:** 10.1371/journal.pone.0160203

**Published:** 2016-08-05

**Authors:** Mohamed Ramadan El-Jade, Marijo Parcina, Ricarda Maria Schmithausen, Christoph Stein, Alina Meilaender, Achim Hoerauf, Ernst Molitor, Isabelle Bekeredjian-Ding

**Affiliations:** 1 Institute for Medical Microbiology, Immunology and Parasitology (IMMIP), University Hospital Bonn, Sigmund-Freud-Str. 25, D-53105, Bonn, Germany; 2 Institute of Animal Science, Preventive Health Management Group, University of Bonn, Katzenburgweg 7–9, D-53115, Bonn, Germany; 3 Division of Microbiology, Paul-Ehrlich-Institut, Paul-Ehrlich-Str. 51–59, D-63225, Langen, Germany; Cornell University, UNITED STATES

## Abstract

Rapid detection and reporting of third generation cephalosporine resistance (3GC-R) and of extended spectrum betalactamases in *Enterobacteriaceae* (ESBL-E) is a diagnostic and therapeutic priority to avoid inefficacy of the initial antibiotic regimen. In this study we evaluated a commercially available chromogenic screen for 3GC-R as a predictive and/or confirmatory test for ESBL and AmpC activity in clinical and veterinary *Enterobacteriaceae* isolates. The test was highly reliable in the prediction of cefotaxime and cefpodoxime resistance, but there was no correlation with ceftazidime and piperacillin/tazobactam minimal inhibitory concentrations. All human and porcine ESBL-E tested were detected with exception of one genetically positive but phenotypically negative isolate. By contrast, AmpC detection rates lay below 30%. Notably, exclusion of piperacillin/tazobactam resistant, 3GC susceptible K1+ *Klebsiella* isolates increased the sensitivity and specificity of the test for ESBL detection. Our data further imply that in regions with low prevalence of AmpC and K1 positive *E*. *coli* strains chromogenic testing for 3GC-R can substitute for more time consuming ESBL confirmative testing in *E*. *coli* isolates tested positive by Phoenix or VITEK2 ESBL screen. We, therefore, suggest a diagnostic algorithm that distinguishes 3GC-R screening from primary culture and species-dependent confirmatory ESBL testing by βLACTA^TM^ and discuss the implications of MIC distribution results on the choice of antibiotic regimen.

## Introduction

Penicillins and cephalosporines are among the most common antibiotic substances used in human and veterinary medicine. Despite the availability of alternative classes of antibiotics up to today the betalactams are first choice substances due to their high efficacy. However, widespread use of betalactams has led to the emergence of betalactam resistance in both Gram positive and Gram negative bacteria. Enzmyes termed “βtermed “erm” cleave the βhe ave “ermed “es termed “myes termethereby prevent their interference with the transpeptidase activity of the tidase ase h hydrolysis and [1]. Over the last decades point mutations in the " \o "Witte W, 20have changed the active site and extended the substrate spectrum [2,3,4]. The arising extended-spectrum beta-lactamases (ESBL) are not only able to hydrolyze narrow-spectrum antibiotics such as penicillins and first and second generation cephalosporins but also inactivate broad-spectrum antibiotics such as aztreonam and third, fourth and fifth generation cephalosporins [5,6]. The spread of these enzymes is facilitated by their encoding on plasmids and represents the major cause for the increased resistance to broad-spectrum esistance ncoding on in enterobacteriaceae [6]. Therapeutic failure of first line antibiotics due to production of ESBL is associated with prolonged hospitalization, increased patient mortality and increased medical costs [7,8,9,10,11].

The methods routinely used for detection of ESBL in clinical isolates are mostly based on phenotypical diagnosis of ESBL involving bacterial culture in the presence or absence of antibiotics and betalactamase inhibitors. These include selective media, ESBL screening algorithms in automated susceptibility testing such as VITEK2 or Phoenix and confirmatory testing with discs or E-test stripes containing betalactam antibiotics with and without supplementation of betalactamase inhibitors [12]. Although genetic proof of ESBL is most convincing, due to the high cost and only few CE-certified commercial assays available, most microbiological laboratories do not routinely use molecular tests for ESBL detection. Nevertheless, false positive and false negative results in automated ESBL screening algorithms require additional confirmatory testing, which is time consuming.

Lately, both EUCAST and CLSI guidelines have lowered their breakpoints for cephalosporine susceptibility testing and switched to the paradigm that results obtained should be reported as measured in the microbiology laboratory [13,14,15,16]. The formerly employed interpretative approach corrected all cephalosporines tested as susceptible to resistant if the isolate was tested positive for ESBL. With the newly defined lower and more sensitive break points this is no longer viewed as necessary [14,17].

It is, however, an ongoing matter of debate whether the presence of ESBL could lead to *in vivo* inefficacy of betalactam antibiotics, in particular third generation cephalosporines (3GC), despite *in vitro* susceptibility of the infecting strains [18,19,20,21]. Nevertheless, confidence in the *in vitro* susceptibility results could open new therapeutic options and reduce the use of broad-spectrum reserve antibiotics such as carbapenems [22,23,24]. In views of the rapid emergence of carbapenemases any therapeutic alternative is an option to be taken under serious consideration.

Obviously, therapeutic decisions based on *in vitro* testing must apply the necessary caution until clinical studies provide sufficient evidence for the efficacy of 3GC in infections with ESBL-E. It is, therefore, an important duty for the microbiologist to provide as much information as possible, i.e. perform ESBL testing of suspected isolates. Since the routine methods currently employed require an additional 18–24 hours for definite diagnosis of ESBL activity, the development of faster methods for reliable prediction of ESBL activity is a diagnostic challenge and a therapeutic priority.

In this study we evaluated a rapid commercially available test for its prediction of ESBL expression in *enterobacteriaceae* cultured from patient materials and pigs. The principle of the ßrom p^TM^ test is based on the cleavage of the substrate HMRZ-86*, a chromogenic cephalosporine [25,26]. This substrate, initially yellow, turns red in the presence of mogenic cephalosporine in n in sion in d-spectrum-beta-lactamase, AmpC, and Carbapenemase issues<linases (e.g. SHV-1, TEM-1) but processed by ESBL, acquired AmpC and carbapenemases (KPC and metallobetalactamases) [26,27]. Thus, the βLACTA^TM^ test offers a rapid method for the identification of strains with resistance to third-generation cephalosporins (3GC-R), e.g. if loss in susceptibility is due to the production of β-lactamases. In the present study we evaluated the utility of this assay as an ESBL confirmatory test in the routine clinical microbiology laboratory.

## Material and Methods

### Bacterial isolates

A total of 245 strains of *Enterobacteriaceae* were retrospectively analyzed. 173 members of the 200 bacterial isolates used for evaluation were collected from patient samples in the routine microbiology laboratory of the University Hospital Bonn from July 2012 to June 2014. The remaining 72 isolates were derived from fecal swabs obtained from pigs subjected to a hygiene monitoring program between June and September 2012 [28]. Species identification was performed with VITEK-MS (bioMh VITx S.A., Nuertingen, Germany). Enterobacteriaceae isolates were pre-selected based on VITEK2 susceptibility results compatible with an ESBL phenotype, growth on ESBL screening agar (ChromID^TM^, bioMmIDing S.A.) selective agar or unbiased collection from single pathogen urinary tract infections with *E*. *coli* or *Klebsiella* spp. over a time period of two weeks. Replicates from the same patient were excluded from the analysis.

### Susceptibility testing and detection of ESBL-E

VITEK2 (bioMK2 (bi S.A., NS.A., Nu, Germany) AST-N214 (REF 413064) and Phoenix 100 (Becton Dickinson, Heidelberg, Germany) UNMIC/ID-87 panel (REF 448771) were used for automated antibiotic susceptibility testing using EUCAST criteria.

Several methods were used in parallel to detect ESBL positive bacterial isolates: culture on ChromID^TM^, bioMoMD on S.A., Nuertingen,Germany) selective agar, VITEK2 (bioMioMivx S.A., NS.A., NN, Germany) and Phoenix 100 (Becton Dickinson, Heidelberg, Germany) ESBL for screens. Third generation cephalosporine resistance (3GC-R) was further screened by βLACTA^TM^ test following the manufactureridelberg, Ge(Bio-Rad, Marnes-la-Coquette, France).

For molecular typing bacterial DNA was isolated using UltraClean^®l^Microbial DNA Isolation Kit (MO BIO Laboratories, Carlsbad, California, USA). The PCR was carried out using the PN-Mix (GenID®eGmbH, Strassberg, Germany) and Taq DNA Polymerase (Thermo Fisher Scientific Inc., Waltham, Massachusetts, USA) on a Labcycler (SensoQuest GmbH, GGmbH, Gs, Germany). Reverse hybridization was performed using the respective biotinylated amplicons using the protocol from GenID^®e^GmbH, Strarberg, Germany with sequence-specific oligonucleotides for betalactamases and controls immobilized on nitrocellulose membranes.

In those isolates tested negative in the molecular ESBL screen ESBL activity was confirmed using the disc diffusion method using AmpC&ESβmpC&EScreen ESBL activCefpodoxim ESpodoxim ESESBL activity was conDiagnostica GmbH) and E-Test ESBL from bioMH) and S.A.

### Statistics

Statistical analysis was performed using iWork, Numbers software version 3.5.3 (Apple Inc., Cupertino, CA, USA). Sensitivity and specificity were calculated as true positive / total positive and true negative / total negative.

## Results

### Positive βLACTA^TM^ results correlate with cefotaxime and cefpodoxime resistance

In the present study we evaluated the βLACTA^TM^ test for detection and confirmation of ESBL. To this end we used a collection of 245 *Enterobactericeae* (170 *E*. *coli*, 58 *Klebsiella* spp, 17 other species) with either negative or suspected ESBL, AmpC or K1 activity based on VITEK2 analysis (Table 1). The isolates were derived from hospitalized patients or from pigs screened for ESBL-E on farms in the nearby region. Among these isolates 72.2% (177/245) were tested as positive for 3GC-R by βLACTA^TM^, 26.5% (65/245) were negative and 1.2% (3/245) of test results were non-interpretable (Table 2). Testing of samples was fast (2 to 5 minutes) and easy-to-handle and could, therefore, represent an interesting alternative to conventional ESBL testing.

**Table 1:** Species-specific distribution of ESBL, AmpC, K1 and KPC in this study.

		ESBL		AmpC		K1*	
Species	#	pos	neg	pos	neg	pos	neg
*Escherichia coli*	170	122	48	4	166	5	165
*Klebsiella pneumoniae*	40	31**	9	1	39	4	36
*Klebsiella oxytoca*	18	2	16	0	18	15	3
*Enterobacter cloacae*	4	0	4	2	2	0	4
*Enterobacter aerogenes*	2	0	2	1	1	0	2
*Serratia marcescens*	3	0	3	2	1	0	3
*Proteus mirabilis*	3	0	3	2	1	0	3
*Morganella morganii*	1	0	1	1	0	0	1
*Citrobacter freundii*	1	1	0	0	1	0	1
*Pantoea agglomerans*	1	1	0	0	1	0	1
*Kluyvera cryocrescens*	1	1	0	0	1	0	1
*Citrobacter amalonaticus*	1	0	1	0	1	0	1
**Total**	**245**	158	87	13	232	24	221

* Piperacillin/Tazobactam-resistant (K1- suspicious) isolates

** one strain also contained KPC

**Table 2:** βLACTA^TM^, VITEK2 and PHOENIX100 results in the strain collective.

		VITEK2			PHOENIX100			βLACTA		
Species	#	pos	neg	n.i.	pos	neg	n.i.	pos	neg	n.i.
*Escherichia coli*	170	125	42	3	125	45	0	123	47	0
*Klebsiella pneumoniae*	40	30	5	5	25	15	0	33	5	2
*Klebsiella oxytoca*	18	16	1	1	13	4	1	15	3	0
*Enterobacter cloacae*	4	0	1	3	1	3	0	2	2	0
*Enterobacter aerogenes*	2	0	0	2	0	2	0	0	1	1
*Serratia marcescens*	3	0	0	3	0	3	0	0	3	0
*Proteus mirabilis*	3	0	0	3	0	3	0	1	2	0
*Morganella morganii*	1	0	0	1	0	1	0	0	1	0
*Citrobacter freundii*	1	1	0	0	1	0	0	1	0	0
*Pantoea agglomerans*	1	0	0	1	0	1	0	1	0	0
*Kluyvera cryocrescens*	1	0	0	1	0	1	0	0	1	0
*Citrobacter amalonaticus*	1	0	0	1	0	1	0	1	0	0
**Total**	**245**	172	49	24	165	79	1	177	65	3

pos: positive, neg: negative, n.i.: non-interpretable

**Fig 1:**
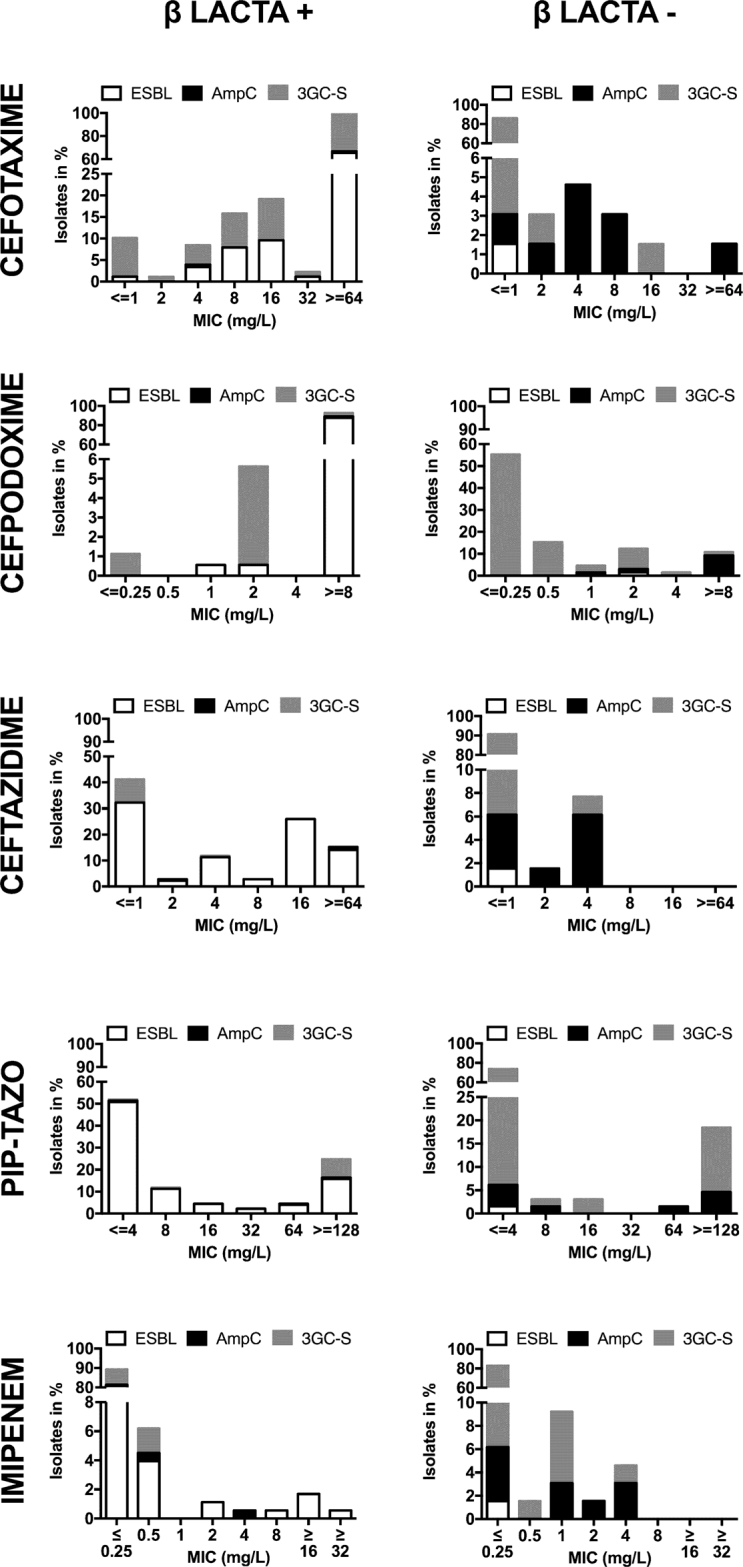
βLACTA^TM^ results in regards to distribution of MICs and ESBL or AmpC. The graphs depict the MIC of cefotaxime (A), cefpodoxime (B), ceftazidime (C), piperacillin/tazobactam (D) and imipenem (E) in the positive (left) and negative (right) βLACTA^TM^ fractions. Information on ESBL activity (white), AmpC expression (black) and 3GC susceptibility (3GC-S, grey) is provided.

The βLACTA^TM^ test is based on chromogenic detection of 3GC-R. To evaluate the method we correlated the minimal inhibitory concentrations (MIC) obtained by VITEK2 analysis with positive and negative βLACTA^TM^ results. The results showed that the fraction of isolates tested positive by βLACTA^TM^ had high MIC values for cefotaxime and cefpodoxime. 66.7% had a MIC for cefotaxime ≥64 mg/L and 89.8% were categorized as resistant to cefotaxime according to EUCAST breakpoints, e.g. cefotaxime MIC >2 (Fig 1). 92.7% of βLACTA positive isolates had a MIC of ≥8 mg/L for cefpodoxime and 98.3% were classified as resistant according to EUCAST breakpoints (MIC >1) (Fig 1). On the contrary, the negative fraction displayed low MIC values for cefotaxime (86.2% susceptible according to EUCAST breakpoints, e.g. ≤1 mg/L) and a variable MIC distribution for cefpodoxime with 75.4% susceptible and 24.6% resistant isolates (Fig 1).

Further analyses revealed that there was no obvious correlation with MIC for ceftazidime, e.g. isolates with MIC ≤ 1 mg/L were found in both fractions. MIC >4 mg/L, however, were confined to the positive fraction (Fig 1). Similarly, we observed no correlation with piperacillin/tazobactam MIC values in any of the fractions, e.g. MIC ≤ 8 and ≥6 were found in both fractions (Fig 1). Lastly, βLACTA^TM^ fractions did not differ in the MIC for imipenem (Fig 1). Altogether, the βLACTA^TM^ results showed the clearest discrimination between cefotaxime susceptible and resistant isolates. Automated susceptibility testing with Phoenix^TM^ provided comparable results (S1 Fig).

### Reliable detection of ESBL with βLACTA^TM^

Next, we assessed whether the βLACTA^TM^ assay could be used to detect ESBL as suggested by the manufacturer. To this end we analyzed the correlation of βLACTA^TM^ test results with ESBL genotyping by PCR. Among the isolates tested positive with the βLACTA^TM^ assay 156 (88.1%) were ESBL positive (TEM (1), SHV (3), CTX-M (147); 5 isolates were double positive: CTX-M+SHV+ (3), CTX-M+TEM+ (1) and KPC+SHV+ (1)) and 21 (11.9%) were negative by molecular testing. In these negative isolates, phenotypical ESBL and AmpC activity were analyzed by E-test and disc diffusion. One isolate displayed ESBL-activity, 3 were AmpC positive and 17 were negative in regards to both.

The 65 isolates of the βLACTA^TM^ negative fraction as well as the 3 non-interpretable results were all ESBL negative by PCR except for one CTX-M positive isolate. This isolate lacked ESBL activity in E-test and phenotypic disc diffusion tests. The PCR negative fraction contained 10 AmpC+ isolates and 58 isolates tested negative for AmpC and ESBL by disc diffusion test and E-test.

Of note, when using the manufacturer test and and ion 171 isolates positive for either ESBL, AmpC or KPC were βLACTA^TM^ positive (93.6%), 9 (5.3%) were negative and 2 (1.1%) non-interpretable. This resulted in an overall sensitivity of 94.7% for undifferentiated detection of ESBL, AmpC and/or KPC. Among the 74 isolates genotypically and phenotypically negative for ESBL, AmpC and KPC 56 (75.7%) were tested βLACTA^TM^ negative, 17 (23%) positive and 1 (1.3%) non-interpretable, providing a specificity of 76.7%. When limiting our analysis to ESBL prediction we found that the sensitivity was 99.4% and specificity 76.2%, e.g. from 158 ESBL positive isolates 157 were βLACTA^TM^ positive (99.4%) and one (0.6%) was negative, and, from 84 ESBL negative isolates 64 (76.2%) were βLACTA^TM^ negative, 20 (23.8%) βLACTA^TM^ positive and 3 (3.6%) results were non-interpretable.

### βLACTA results in AmpC-expressing strains

We, next, wanted to assess whether the βLACTA^TM^ test can identify AmpC producers as indicated by the manufacturer. However, in our strain collection the βLACTA testing only detected 3 out of 13 AmpC-expressing strains (Table 1). Thus, the sensitivity and specificity for AmpC detection were low, e.g. 27.3% and 24.7%, respectively.

### Sensitivity of the βLACTA^TM^ test is superior to that of selective ESBL agar plates

Screening of patients for ESBL-E carriage is routinely performed with selective media containing cephalosporines. Comparison of βLACTA^TM^ results to growth on ChromID^TM^ ESBL agar showed comparable sensitivities for detection of ESBL-E, e.g. 99.4% and 100%, respectively. The specificity was higher for βLACTA^TM^, e.g. 76.2% versus 10.3% for ChromID^TM^ ESBL agar.

### The specificity of the βLACTA^TM^ test is superior to that of VITEK2 and Phoenix ESBL screens

In infection, ESBL activity is usually suspected based on an ESBL screening algorithm employed during automated susceptibility testing of the clinical isolate. We, thus, compared βLACTA^TM^ testing to the ESBL screens provided on VITEK2 and Phoenix systems. Among the βLACTA^TM^ positive fraction 91.5% (162/177) of isolates were ESBL positive in the VITEK2 screen (Table 2), 1.7% (3/177) were negative and 6.8% (12/177) not interpretable by VITEK2. In the βLACTA^TM^ negative fraction 11 isolates were positive, 10 not interpretable and only 44 were negative by VITEK2 analysis. Sensitivity (98.7%) and specificity (64.8%) of the VITEK2 ESBL screen were, thus, lower than those achieved by βLACTA^TM^. Despite lower sensitivity (90.5%) the Phoenix ESBL test was superior to the VITEK2 ESBL screen with 74.4% specificity for ESBL-E (Table 2). However, it remained below the specificity level of βLACTA^TM^.

### Species-specific differences in ESBL detection with βLACTA^TM^

A more detailed analysis revealed that the ESBL-negative isolates tested false positive by βLACTA^TM^ mainly belonged to the *Klebsiella* spp., while the majority of isolates in the βLACTA^TM^ positive fraction were *E*. *coli*. The βLACTA^TM^ negative fraction displayed a higher diversity in regards to species variation (Table 2).

Since false positive results were mainly obtained with *Klebsiella* spp. isolates we compared the βLACTA^TM^ results of *E*. *coli* and *Klebsiella* spp. isolates. Differential analysis of the *Klebsiella* spp. isolates revealed that among the 48 βLACTA^TM^ positive isolates only 33 were ESBL positive and 15 were ESBL negative (Table 3). 8 isolates were concordantly negative for βLACTA^TM^ and ESBL (Table 3). For detection of ESBL, this provided a sensitivity of 100% but a specificity of only 34.8% when testing *Klebsiella* spp. (Table 3). Our analysis of the *E*. *coli* isolates showed that among the strains tested 122/170 were ESBL positive and 48/170 were ESBL negative (Table 1). In this subcategory of isolates all isolates were classified correctly by βLACTA^TM^ with one exception, an AmpC+ strain, which was tested positive. This resulted in 100% sensitivity and 97.9% specificity for ESBL detection in the *E*. *coli* strains tested (Table 3).

**Table 3:** Sensitivity and specificity for βLACTA^TM^ for 3-GCR, ESBL and AmpC in *Enterobacteriaceae* and for ESBL detection in *E*. *coli* and *Klebsiella* spp.

	# n.i.	# false / total positive	# false / total negative	Sensitivity (%)	Specificity (%)
***Entero-bacteriaceae***					
3GC-R	3 (2 3GC-R)	17/177	9/65	94.7	76.7
ESBL+	3 (3 ESBL-)	20/177	1/65	99.4	76.2
AmpC	3 (2 AmpC+)	174/177	8/65	27.3	24.7
***E*. *coli***					
ESBL+	0	1/123	0/47	100	97.9
***Klebsiella spp*.**					
ESBL+	2 (2 ESBL-)	15/48	0/8	100	34.8

n.i. = non-interpretable

Comparison with the species-specific reliability of VITEK2 and Phoenix in regards to ESBL prediction in *E*. *coli* our results showed that VITEK2 and Phoenix reached sensitivities of 100% and 97.5%, respectively, and a specificity of 87.5%, which lay markedly below that obtained by βLACTA^TM^ (Table 4). For *Klebsiella* species sensitivities where lower than those achieved with the βLACTA test, e.g. 93.3 and 69.7%, respectively (Table 4). The specificity of ESBL detection in VITEK2 was lower than that obtained by the βLACTA^TM^ test, e.g. 18.2% (Table 4). However, with *Klebsiella* spp. the Phoenix analysis was slightly better than the βLACTA^TM^ test with 37.5% compared to 34.8% specificity (Table 4).

**Table 4:** Sensitivity and specificity obtained by VITEK2 and Phoenix100 ESBL algorythms in *Enterobacteriaceae*, *E*. *coli* and *Klebsiella* spp.

	# n.i.	# false / total positive	# false / total negative	Sensitivity (%)	Specificity (%)
**VITEK2**					
*Enterobacteriaceae*	24 (8)	25/173	2/48	**99**	**64.8**
*E*. *coli*	3 (3)	6/125	0/42	**100**	**87.5**
*Klebsiella spp*.	6 (3)	18/46	2/6	**93.3**	**18.2**
**Phoenix 100**					
*Enterobacteriaceae*	1 (0)	22/165	15/79	**90.5**	**74.4**
*E*. *coli*	0	6/125	3/45	**97.5**	**87.5**
*Klebsiella spp*.	1 (0)	15/38	10/19	**69.7**	**37.5**

n.i. = non-interpretable (# of n.i. ESBL+ isolates)

### *Klebsiella* K1 isolates reduce βLACTA specificity

More profound analysis revealed that from the 17 false positive βLACTA^TM^ results in the total enterobacteriaceae collective 14 could be attributed to *Klebsiella* spp. isolates resistant to both piperacillin/tazobactam (MIC ≥128 mg/L) and cefpodoxime (two with non-interpretable AmpC test) and to one piperacillin/tazobactam resistant but 3GC susceptible *Klebsiella* spp. isolate. The residual two false positive results were found in a piperacillin/tazobactam susceptible (MIC = 8), cefpodoxime and cefotaxime resistant *Enterobacter cloacae* isolate and a 3GC susceptible *Citrobacter amalonaticus* isolate.

Upon exclusion of phenotypical K1+ *Klebsiella* spp. isolates we achieved a sensitivity and specificity of 100% for ESBL-E detection. However, the absolute number of piperacillin/tazobactam susceptible *Klebsiella* spp. isolates was low (3 ESBL-E, 3 non-ESBL-E). Moreover, 4 piperacillin/tazobactam resistant *Klebsiella* spp. and 5 piperacillin/tazobactam resistant ESBL/AmpC-negative *E*. *coli* isolates were tested negative by βLACTA^TM^, indicating that, in turn, βLACTA^TM^ test does not necessarily react with phenotypical K1+ isolates.

Taken together, the results obtained demonstrated that upon exclusion of suspected K1+ *Klebsiella* spp. isolates the βLACTA^TM^ test displays high reliability for prediction of true ESBL-E.

## Discussion

Presently, EUCAST and CLSI guidelines recommend reporting of betalactam susceptibility testing results as measured omitting secondary ESBL testing. However, *in vitro* susceptibility to cephalosporines might not always predict their *in vivo* efficacy in infections with *Enterobacteriaceae* expressing extended spectrum betalactamases (ESBL-E) and bears the risk of recurrence or prolonged and more severe disease [29]. This therapeutic risk has raised an intense debate among microbiologists and infectious disease specialists [18,21] and defined a new need for the detection of ESBL in the routine diagnostic laboratory. The study presented here was performed on this background.

The data obtained show that the chromogenic 3GC-R test applied in this study might represent an additional option for screening for 3GC-R in primary cultures, in particular *E*. *coli* isolates. For *Klebsiella* spp. prediction of 3GC-R was less reliable because of false positive testing of K1+ *Klebsiella* strains (Table 3). Furthermore, it was demonstrated that the βLACTA test can be used for prediction of ESBL activity in *Enterobacteriaceae*. While the sensitivity and specificity for ESBL was excellent in *E*. *coli*, regardless of their source, there were insecurities in regards to ESBL prediction in *Klebsiella* species (Table 3). This corroborates and expands the findings of Morosini et al. who reported 97.5% true positive results with the βLACTA^TM^ test in their strain collection [30].

Notably, the sensitivities achieved by the VITEK2 and Phoenix100 ESBL algorithms were comparable to the βLACTA^TM^ test in *E*. *coli* isolates (Tables 3 and 4). However, the specificities obtained by these instruments were clearly lower when compared to the βLACTA^TM^. In *Klebsiella* isolates the sensitivity of the βLACTA^TM^ test for ESBL-E was comparable to that in *E*. *coli* (100%) and sensitivity of VITEK2 was only slightly lower (93.3%), at the cost of very low specificity; Phoenix analyses provided a slightly higher specificity but a number of false negative results. Altogether, the accuracy of the βLACTA^TM^ test proved to be higher than that provided by the two automated susceptibility testing systems evaluated. It could, therefore, serve as a complementary tool in the detection and confirmation of ESBL-E.

**Fig 2:**
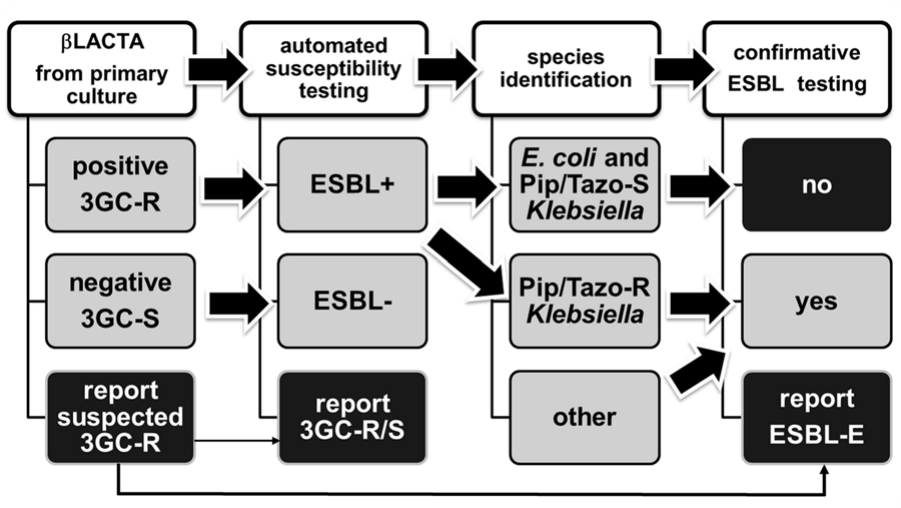
Algorythm for diagnostic use of a chromogenic 3GC-R test in reporting of ESBL-E. The diagram depicts the integration of the chromogenic 3GC-R test in early detection of 3GC-R in primary cultures (left): suspected 3GC-R (βLACTA^TM^ or comparative test positive (A)) is confirmed by automated susceptibility testing (AST) (B) results. It further expands the use of the chromogenic 3GC-R test to ESBL confirmatory testing: if AST (B) delivers positive result for 3GC-R and ESBL screen and isolates are *E*. *coli* or piperacillin/tazobactam susceptible (Pip/Taz-S) *Klebsiella* spp. (C) the βLACTA^TM^ test can be used for confirmation of ESBL (D). Reliability of the βLACTA^TM^ test is decreased in piperacillin/tazobactam resistant (Pip/Taz-R) *Klebsiella* spp. and, thus, not recommended. Other *Enterobacteriaceae* species need to be evaluated.

Based on our findings we propose that with *E*. *coli* isolates positivity of βLACTA^TM^ together with 3CG-R in the antimicrobial susceptibility testing omits the requirement for additional specific ESBL testing. We favor implementation of a step-wise algorithm for its use in the daily routine. Fig 2 depicts a model algorithm for the use of βLACTA^TM^ on primary *Enterobacteriaceae* isolates. This algorithm can easily be applied on blood culture and urine isolates. In our hands, it enables the laboratory to report suspected 3GC-R with a sensitivity of 89.7% at an early time point, thus, providing therapeutically relevant information for an early adjustment of antibiotic therapy.

Recently, Gallah et al. reported 94% sensitivity and a specificity of 100% for detection of ESBL-E by βLACTA^TM^ in enterobacteriaceae isolates from urine specimen [31]. However, the present study demonstrates that phenotypical K1+ *Klebsiella* isolates diminished the accuracy of ESBL detection and ESBL confirmatory testing can, therefore, not be applied to these isolates. Thus, in *Klebsiella* species, ESBL activity can only be predicted in piperacillin/tazobactam susceptible isolates and additional analyses are necessary to confirm ESBL (and AmpC) activity in piperacillin/tazobactam resistant isolates. Moreover, method validation and routine surveillance in the local patient population should confirm the lack of interference of K1+ *E*. *coli* isolates with the βLACTA^TM^ test result before test implementation.

The chromogenic substrate HMRZ-86 is a cephalosporin originally described to be specifically cleaved by class A ESBL and class D oxacillinases, but not by penicillinases [25,26,27]. It was further suggested that addition of chelating agents can prevent activity of class B metallobetalactamases or class C cephalosporinases, including AmpC on HMRZ-86 [25,26,27]. Our results confirm that HMRZ-86 can serve as a chromogenic substrate for detection of ESBL. On the contrary, we could not confirm detection of class D oxacillinases by βLACTA^TM^ due to lack of OXA-48 positive ESBL negative *Klebsiella* and *E*. *coli* strains. Indeed, OXA-48-expressing strains were previously found to co-produce ESBL in 75% [32], impeding us to distinguish OXA-48 from ESBL activity in βLACTA^TM^ testing. Similarly, we could not distinguish KPC from ESBL activity in a *K*. *pneumoniae* strain bearing both KPC and SHV-ESBL. Lastly, the commercially available βLACTA test did not deliver reliable results for AmpC activity (Table 1), albeit this is suggested by the manufacturer.

Based on its chemical structure, which resembles cefotaxime, it was not surprising that the results obtained with HMRZ-86 correlated best with cefotaxime MICs, a cephalosporine previously reported to serve as a reliable predictor of ESBL- and AmpC-conferred resistance in *E*. *coli* and *Klebsiella pneumoniae* [33]. In this study, βLACTA^TM^ positivity correlated with ESBL detection by PCR and phenotypic ESBL detection. However, βLACTA positive testing and detection of ESBL did neither correlate with high MICs for ceftazidime nor with piperacillin/tazobactam resistance (Fig 1), whose clinical efficacy is currently being reevaluated for therapy of ESBL-E infections [11,16,21,22,24,34,35]. Notably, in VITEK2 analysis only 48.1% of ESBL-E displayed *in vitro* resistance to ceftazidime according to EUCAST criteria, e.g. MIC >4, while 39.2% of ESBL-E and 87.4% non-ESBL-E displayed MICs <4 (Fig 1) albeit an earlier study that the βLACTA^TM^ test was useful in discriminating ceftazidime-susceptible from tor of ESBL*Pseudomonas aeruginosa* isolates [36]. Furthermore, only 24.7% of ESBL-E (and 10.7% of *E*. *coli* ESBL-E) were resistant to piperacillin/tazobactam *in vitro*, e.g. displayed MICs >16 (EUCAST criteria) (Fig 1), while 70.3% of ESBL-E (86.9% of *E*. *coli* ESBL-E) were susceptible to piperacillin/tazobactam *in vitro*. Non-ESBL isolates were found in both the piperacillin/tazobactam susceptible and resistant (i.e. K1+ *Klebsiella* spp.) fractions (Fig 1). Thus, due to lack of correlation the test provides no additional information in regards to the prediction of the *in vivo* efficacy of ceftazidime and/or piperacillin/tazobactam in the patient.

Altogether, our data determine that the βLACTA^TM^ test is a fast and reliable method to predict 3GC-R in *Enterobacteriaceae* and ESBL in *E*. *coli* isolated from humans and pigs. When used in combination with automated susceptibility testing it has a complementary function and can be used to predict ESBL in *E*. *coli* and preselected *Klebsiella* strains.

## Supporting Information

S1 Fig Correlation of βLACTA^TM^ with automated susceptibility testing using Phoenix^TM^. The graph shows positive (left) and negative (right) βLACTA^TM^ results and the respective distribution of MIC (number of isolates for each MIC value) of cefotaxime (upper panel), ceftazidime (middle), piperacillin/tazobactam (lower panel) susceptibility testing obtained by Phoenix^TM^ analysis.
(PDF)
