# Correction: ESBL Detection: Comparison of a Commercially Available Chromogenic Test for Third Generation Cephalosporine Resistance and Automated Susceptibility Testing in *Enterobactericeae*

**DOI:** 10.1371/journal.pone.0198959

**Published:** 2018-06-07

**Authors:** Mohamed Ramadan El-Jade, Marijo Parcina, Ricarda Maria Schmithausen, Christoph Stein, Alina Meilaender, Achim Hoerauf, Ernst Molitor, Isabelle Bekeredjian-Ding

There are errors in the fourth and fifth sentences of the Introduction. The correct sentences are: Enzymes termed “β-lactamases” cleave the β-lactam ring through hydrolysis and thereby prevent their interference with the transpeptidase activity of the “penicillin-binding proteins” [1]. Over the last decades point mutations in the β-lactamase genes changed the active site and extended the substrate spectrum [2,3,4].

There is an error in the penultimate sentence of the first paragraph in the Introduction. The correct sentence is: The spread of these enzymes is facilitated by their encoding on plasmids and represents the major cause for the increased resistance to broad-spectrum β-lactam antibiotics on in *Enterobacteriaceae* [6].

There is an error in the second sentence of the final paragraph of the Introduction. The correct sentence is: The principle of the βLACTA^TM^ test is based on the cleavage of the substrate HMRZ-86*, a chromogenic cephalosporine [25,26].

The third and fourth sentences of the final paragraph of the Introduction contain errors and have been incorrectly combined. The correct sentences are: This substrate, initially yellow, turns red in the presence of ß-lactamases that confer resistance to 3GC. Notably, HMRZ-86 is not hydrolyzed by acquired penicillinases (e.g. SHV-1, TEM-1) but processed by ESBL, acquired AmpC and carbapenemases (KPC and metallobetalactamases) [26,27].

In the Materials and Methods section, the fourth sentence under the “Bacterial isolates” heading is incorrect. The correct sentence is: Species identification was performed with VITEK-MS (bioMérieux S.A., Nuertingen, Germany).

In the Materials and Methods section, in the fifth sentence under the “Bacterial isolates” heading the ESBL screening agar should be specified as: ChromID^TM^, bioMérieux S.A..

In the first sentence of the second paragraph under the “Susceptibility testing and detection of ESBL-E” heading of the Material and Methods section the VITEK2 manufacturer should be specified as: bioMérieux S.A., Nürtingen, Germany.

In the second sentence of the second paragraph under the “Susceptibility testing and detection of ESBL-E” heading of the Material and Methods section the ChromID^TM^ selective agar and the VITEK2 should both be labelled as products from: bioMérieux S.A., Nürtingen, Germany.

The final sentence in the second paragraph under the “Susceptibility testing and detection of ESBL-E” heading of the Material and Methods section is incorrect. The correct sentence is: Third generation cephalosporine resistance (3GC-R) was further screened by βLACTA^TM^ test following the manufacturer´s protocol (Bio-Rad, Marnes-la-Coquette, France).

Throughout the third paragraph under the “Susceptibility testing and detection of ESBL-E” heading of the Material and Methods section, incorrect symbols and characters follow the registered trademark symbol. The correct sentences are: For molecular typing bacterial DNA was isolated using UltraClean^®^ Microbial DNA Isolation Kit (MO BIO Laboratories, Carlsbad, California, USA). The PCR was carried out using the PN-Mix (GenID^®^ GmbH, Strassberg, Germany) and Taq DNA Polymerase (Thermo Fisher Scientific Inc., Waltham, Massachusetts, USA) on a Labcycler (SensoQuest GmbH, Göttingen, Germany). Reverse hybridization was performed using the respective biotinylated amplicons using the protocol from GenID^®^ GmbH, Straßberg, Germany with sequence-specific oligonucleotides for betalactamases and controls immobilized on nitrocellulose membranes.

The final sentence under the “Susceptibility testing and detection of ESBL-E” heading of the Material and Methods is incorrect. The correct sentence is: In those isolates tested negative in the molecular ESBL screen ESBL activity was confirmed using the disc diffusion method using AmpC&ESβL Detection Discs and Cefpodoxim ESβL ID Disc Set (both from Mast Diagnostica GmbH) and E-Test ESBL from bioMérieux S.A..

The fourth sentence of the penultimate paragraph of the Discussion section is incorrect. The correct sentence is: Notably, in VITEK2 analysis only 48.1% of ESBL-E displayed *in vitro* resistance to ceftazidime according to EUCAST criteria, e.g. MIC >4, while 39.2% of ESBL-E and 87.4% non-ESBL-E displayed MICs <4 (Fig 1) albeit an earlier study that the βLACTA^TM^ test was useful in discriminating ceftazidime-susceptible from resistant Pseudomonas aeruginosa isolates [36].

The fourth sentence of the penultimate paragraph of the Discussion section is incorrect. The correct sentence is: Notably, in VITEK2 analysis only 48.1% of ESBL-E displayed *in vitro* resistance to ceftazidime according to EUCAST criteria, e.g. MIC >4, while 39.2% of ESBL-E and 87.4% non-ESBL-E displayed MICs <4 (Fig. 1) albeit an earlier study that the βLACTA^TM^ test was useful in discriminating ceftazidime-susceptible from–resistant Pseudomonas aeruginosa isolates [36].

## References

[1] El-Jade MR, Parcina M, Schmithausen RM, Stein C, Meilaender A, Hoerauf A, et al (2016) ESBL Detection: Comparison of a Commercially Available Chromogenic Test for Third Generation Cephalosporine Resistance and Automated Susceptibility Testing in *Enterobactericeae*. PLoS ONE 11(8): e0160203 https://doi.org/10.1371/journal.pone.0160203 27494134 10.1371/journal.pone.0160203 PMC4975492

