# Supplementary material for: ESBL Detection: Comparison of a Commercially Available Chromogenic Test for Third Generation Cephalosporine Resistance and Automated Susceptibility Testing in Enterobactericeae
Source: PLoS One. 2016 Aug 5;11(8):e0160203. doi: 10.1371/journal.pone.0160203 (PMC4975492; doi:10.1371/journal.pone.0160203)
Supplement: S1 Fig — The graph shows positive (left) and negative (right) βLACTATM results and the respective distribution of MIC (number of isolates for each MIC value) of cefotaxime (upper panel), ceftazidime (middle), piperacillin/tazobactam (lower panel) susceptibility testing obtained by PhoenixTM analysis. (PDF) [file pone.0160203.s001.pdf]

Supporting information S1 Fig.

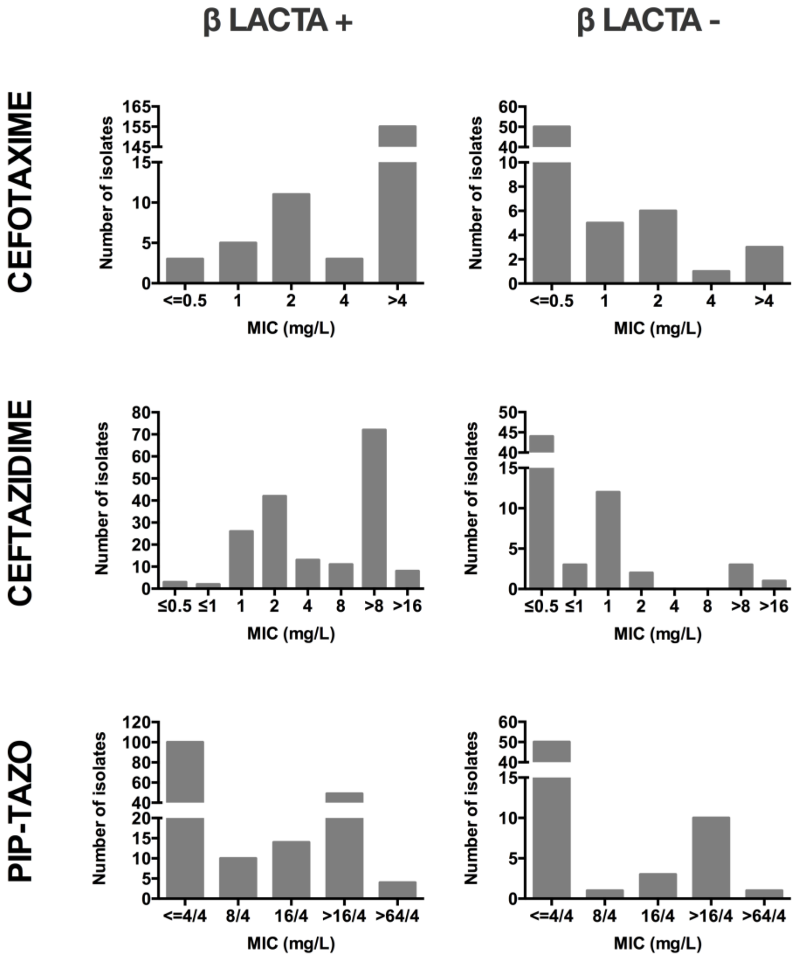

S1 Fig. Correlation of βLACTA™ with automated susceptibility testing using Phoenix™. The graph shows positive (left) and negative (right) βLACTA™ results and the respective distribution of MIC (number of isolates for each MIC value) of cefotaxime (upper panel), ceftazidime (middle), piperacillin/tazobactam (lower panel) susceptibility testing obtained by Phoenix™ analysis.
